# A Study of Cadaveric Skin Graft Harvest and Usage: An Observational Prospective Pilot Study

**DOI:** 10.7759/cureus.69932

**Published:** 2024-09-22

**Authors:** Anup P B, Guruswamy Vishwanath, Chetankumar R Tikar, Swati G Deshpande

**Affiliations:** 1 General Surgery, Military Hospital Bareilly, Bareilly, IND; 2 Resident, General Surgery, INHS, Asvini, Colaba, Mumbai, IND; 3 Plastic Surgery, Dnyandeo Yashwantrao (DY) Patil Medical College Hospital and Research Centre, Pune, IND; 4 Plastic Surgery, Indian Naval Hospital Ship Asvini, Mumbai, IND; 5 Urology, Nair Hospital, Mumbai, IND; 6 General Surgery, Indian Naval Hospital Ship Asvini, Mumbai, IND; 7 Surgery, Jawaharlal Nehru Medical College, Datta Meghe Institute of Higher Education and Research, Wardha, IND

**Keywords:** burn care, burns, cadaveric skin grafts, graft uptake, skin donation, tissue banking, wound management

## Abstract

Background

Burn injuries pose a significant global public health challenge, causing substantial morbidity and mortality, particularly in low- and middle-income countries. Timely and effective wound coverage is critical in treating severe burns to prevent infection, reduce pain, and promote healing. Cadaveric skin grafts (allografts) have become essential in treating extensive burn wounds, serving as temporary biological dressings to prepare the wound bed for autografting. This study aims to comprehensively analyze the process of cadaveric skin graft harvest and usage in a tertiary care setting. It seeks to evaluate the procedures, challenges, and outcomes associated with cadaveric skin grafts, contributing to optimizing burn care practices and improving patient outcomes.

Methods

This observational study was conducted at a tertiary care hospital and burns center with a skin bank, involving 44 cadaveric skin graft harvests and 27 applications between July 1, 2011, and June 30, 2013. The study focused on prospective donors and recipients needing cadaveric skin grafts. Inclusion criteria for donors included consent from the next of kin and the absence of infections or septicemia, while exclusion criteria included prolonged post-mortem intervals and medico-legal cases. The procedures adhered to the Euro Skin Bank protocols, encompassing retrieval, processing, storage, and usage. Data were analyzed using Epi-Info version 7.2.1 software, employing descriptive statistics for categorical variables. Ethical clearance was obtained from the university ethical committee, with mandatory written informed consent for skin donation.

Results

Out of 519 deaths in the tertiary care hospital, significant barriers to skin donation included septicemia, skin changes, late reporting, young age, medico-legal issues, and positive viral markers. Notably, 114 (21.97%) of next of kin refused consent. Cadaveric skin was harvested in five (11.36%) cases, with potential donors identified in 78 (15.02%) of deaths. Donors were predominantly older males, with efficient procurement processes ensuring timely harvests. The tertiary burns center facilitated 39 (88.63%) cases of cadaveric skin harvests with a skin bank, either at the donor's home or other hospitals notified to the burns center. Cadaveric skin grafts were applied in 27 cases, primarily for burns, with high graft uptake observed over 10 days. Non-healing ulcers showed 100% graft uptake. The survival rate among burn patients was 20 (74%), with deaths mainly due to sepsis and multi-organ failure. Significant barriers to obtaining consent included a lack of awareness, superstitions, social stigmas, and religious objections.

Conclusion

The study highlights the critical role of cadaveric skin in managing extensive wounds, particularly burns. Despite challenges in obtaining consent and limited donor availability, cadaveric skin grafts effectively prepared wound beds for autografting, contributing to improved patient outcomes. Increasing community awareness and addressing superstitions and social stigmas are essential for improving donation rates.

## Introduction

Burn injuries represent a significant global public health challenge, causing substantial morbidity and mortality, particularly in low- and middle-income countries. Burn injuries result in approximately 180,000 deaths annually, with the vast majority occurring in low-resource settings [1]. In the clinical management of severe burns, timely and effective wound coverage is critical to prevent infection, reduce pain, and promote healing. Cadaveric skin grafts, also known as allografts, have become vital in treating extensive burn wounds, serving as a temporary biological dressing to prepare the wound bed for autografting [2]. Cadaveric skin grafts offer several advantages, including immediate availability, reduced donor site morbidity, and the ability to cover large wound areas, thereby stabilizing the patient and preventing further physiological deterioration [3]. Allografts act as a temporary biological dressing, promoting revascularization and providing a barrier against infection while maintaining a moist wound environment conducive to healing [4]. However, using cadaveric skin grafts is not without challenges, including the risk of disease transmission, immunogenicity, and limited availability due to the scarcity of skin donors [5].

In tertiary burn centers, where managing extensive and complex burn injuries is routine, the procurement, processing, and application of cadaveric skin grafts require a well-coordinated and multidisciplinary approach. This involves meticulous donor selection, stringent screening for transmissible diseases, and adherence to protocols for skin harvest, preservation, and application to ensure the safety and efficacy of the grafts [6]. Advances in tissue banking and graft preservation techniques have significantly improved the viability and shelf-life of cadaveric skin, making it a more reliable option for burn care [7]. Despite these advancements, there is a paucity of comprehensive studies examining the practical aspects of cadaveric skin graft usage in tertiary burn centers, particularly regarding graft harvest techniques, storage protocols, and clinical outcomes. This study aims to address this gap by providing an in-depth analysis of cadaveric skin graft harvest and usage in a tertiary burn center. By evaluating the procedures, challenges, and outcomes associated with cadaveric skin grafts, this research seeks to contribute to optimizing burn care practices and improving patient outcomes.

The primary objective of this study was to thoroughly examine the process of cadaveric skin graft harvest and its subsequent usage for skin grafting in clinical settings. This includes a detailed exploration of various facets of cadaveric skin grafting, encompassing the donation process, harvesting techniques, preservation methods, and practical application. The study aims to provide comprehensive insights into the practical application of cadaveric skin grafts for treating burns, diabetic wounds, and large wounds resulting from trauma. By addressing these aspects, the research seeks to enhance the understanding and implementation of cadaveric skin grafting in clinical practice, ultimately improving patient care and outcomes.

## Materials and methods

Study design

This observational study was conducted at a tertiary care hospital and burn center equipped with a skin bank, encompassing 44 cadaveric skin graft harvests and the application of cadaveric skin grafts on 27 patients between July 1, 2011, and June 30, 2013. The primary aim was to elucidate the processes involved in the donation, harvesting, storage, and usage of cadaveric skin in patients with extensive wounds resulting from burns, diabetes, or trauma, where the patient's skin was either contaminated or unavailable.

Target population

The study focused on prospective donors who had pledged to donate their skin upon death, as well as recipients in need of cadaveric skin grafts. This included individuals who consented to donate skin posthumously, as well as patients with severe wounds requiring temporary wound coverage.

Inclusion criteria

In order to harvest and preserve skin, individuals had to fulfill two requirements: they must have pledged to donate skin in the event of their death, and they must have their relatives or next of kin give consent for posthumous skin grafting. The death must have occurred within six hours, absence of skin and systemic infections such as HIV, HBsAg, and syphilis; death was not due to septicemia, and the age was over 18 years. The criteria for using skin grafts included patients with contaminated or unavailable skin due to burns, diabetes, non-healing ulcers, and trauma.

Exclusion criteria

We excluded individuals from skin harvest and preservation if their death was more than six hours, if their next of kin refused consent for skin grafting, if their donors had skin or systemic infections, if their deaths were due to septicemia, and if they were under the age of 18. We excluded deaths that required a postmortem or a medico-legal case. Patients who refused to accept cadaveric skin grafts due to religious issues were not eligible for skin graft usage.

Methodology

The procedures for skin harvesting, transportation, preservation, and usage adhered to the protocols established by the Euro Skin Bank, a division of the Euro Tissue Bank in Amsterdam. We divided the methodology into four broad categories: retrieval, skin processing in the lab, storage, and usage.

Retrieval

Awareness and educational initiatives included placing posters with contact information throughout the hospital, establishing collaborations with the transplant coordinator and ophthalmology department for combined donations, and conducting sensitization lectures. We maintained a 24/7 hotline for this purpose.

Skin recovery

After receiving information about a potential donor, a recovery team consisting of one medical officer and two medical assistants equipped with pre-sterilized materials proceeded to the location. The materials included shoe covers, sterile work gowns, surgical gloves, a pressure bandage, sponges, sterilizing agents, a dermatome or Humby knife, blades, syringes, blood tubes, sterile solutions, razors, plastic sheets, towels, saline, sterile skin procurement package, a glycerol solution, antibiotics, and necessary instruments like forceps and scissors. We obtained consent for a skin donation and completed the donor screening process. We shaved the donor's skin, cleansed it with betadine or povidone-iodine, and harvested it.

Processing of harvested cadaveric skin

We transported the harvested skin to the lab in a sterile container with 50% glycerol for further processing. The lab used instruments such as a centrifuge, sealer, meshes, shaking incubator, biosafety cabinet, and skin bank fridge. After checking and lubricating the skin, we placed it in 85% glycerol and incubated it for three hours at 33°C. We stored it in a freezer at up to 8°C for four to six weeks until we received serological reports after incubation.

Further processing

We reviewed the serological reports and then further processed the skin in a biosafety cabinet. We prepared, meshed, and stored symmetrical strips in 85% glycerol, ensuring their readiness for use. Patients with burns and non-healing ulcers received the cadaveric skin as a temporary wound cover, secured with staples or sutures following thorough saline washing to eliminate the glycerol.

Usage

Patients with extensive wounds for whom autologous skin grafts were not feasible primarily utilized cadaveric skin. This included cases of severe burns, diabetic ulcers, non-healing ulcers, and trauma. We carefully applied the harvested, processed, and stored skin to the wound sites to provide temporary coverage and promote healing. We analyzed the data using Epi-Info version 7.2.1 (CDC Atlanta) software. We used descriptive statistics, frequency distribution, and proportions for categorical variables.

Ethical clearance

The study was approved by the university ethical committee to which the institute was affiliated. Written informed consent was mandatory for skin donation. We adhered to ethical standards to conduct cadaveric skin donation and usage responsibly and with respect for the donors and their families.

## Results

Cadaveric skin graft harvest

During the study period, the total number of deaths recorded at the tertiary care hospital was 519. Of these deaths, 283 (54.53%) were directly or indirectly attributed to septicemia, rendering these individuals unsuitable for skin donation. Additionally, 31 (5.97%) cases exhibited skin changes such as hyperpigmentation, excoriation, or infections, excluding them from potential donation. Thirteen (2.50%) deaths were either not reported within six hours or exceeded the six-hour post-mortem timeframe, while 26 (5.01%) individuals were below the age of 18 and thus ineligible for donation. Moreover, 39 (7.52%) cases were medico-legal or required a post-mortem examination, and eight (1.54%) cases tested positive for triple viral markers, making them unsuitable for skin graft harvest. Notably, the next of kin in 114 (21.97%) cases refused to consent to skin donation. Consequently, cadaveric skin was harvested in only five (0.96%) cases with the consent of the next of kin. Among the 114 (21.97%) cases without consent, 78 (68.42%) potential donors met the inclusion and exclusion criteria. The highest incidence of potential donors was in January 2012, with eight out of 22 (36.36%) deaths identified as potential donors. Conversely, November 2011 and August 2012 had the lowest incidence rates, at 5/114 (4.54%) and 6/114 (4.76%), respectively. The average incidence of potential donors across the 24 months was 78/519 (15.02%).

Issues in obtaining consent

The study identified several barriers to obtaining consent from the next of kin for cadaveric skin donation. These included a lack of awareness about the benefits and process of skin donation, superstitions surrounding death and body integrity, social stigmas, and religious objections. These factors significantly influenced the willingness of families to consent to skin donation.

Demographics of Donors

Out of the 44 donors, 30 (68.18%) were male and 14 (31.82%) were female. The donors' ages ranged from 41 to 94 years, with the highest proportion (31.81%) falling within the 71-80 years age group, as shown in Table 1. The second-largest group was those aged 61-70, comprising 11 (25%) of the donors, followed by those aged 81-90 at nine (20.45%) donors. The age distribution indicates a predominance of older donors, which is typical given the natural demographic of hospital deaths (Table 1). The timing of graft procurement post-mortem is critical to ensure the viability and quality of the harvested skin. Most donors (14/44 (31.81%) had their skin harvested within three to four hours post-mortem. This was followed by 12/44 (27.28%) within two to three hours and 22.72% within four to five hours. Only a small percentage 1/44 (2.27%) had skin harvested within the first hour after death. This distribution underscores the efficiency of the procurement process, ensuring timely retrieval of viable skin grafts (Table 1). Most donations 39/44 (88.63%) were facilitated by the tertiary burns center with a skin bank, either at the donor's home or other hospitals notified to the burns center. Only 5/44 (11.37%) of the donations were procured directly from the tertiary care hospital. This data highlights the pivotal role of the tertiary burns center in managing and coordinating skin donation and procurement activities (Table 1).

**Table 1:** Demographics of donors

Variables		Donors (n=44)	Percentage (%)
Age (yrs)	40-50	05	11.37%
	51-60	04	9.10%
	61-70	11	25%
	71-80	14	31.81%
	81-90	09	20.45%
	91-100	01	2.27%
Time between death and graft procurement	Within 1 hour	01	2.27%
	1 hr - 2 hrs	04	9.10%
	2 hrs - 3 hrs	12	27.28%
	3 hrs - 4 hrs	14	31.81%
	4 hrs - 5 hrs	10	22.72%
	5 hrs - 6 hrs	03	6.82%
Location	Tertiary burn center with skin bank	39	88.63%
	Tertiary care hospital	05	11.37%

Cadaveric skin graft usage

During the study period, cadaveric skin grafts were used in 27 cases. Among these, 25 (92.59%) applications were for burn patients (Figure 1-6), while two (7.4%) were for non-healing ulcers (Figure 7-11). The graft uptake was monitored on days five, seven, and 10 following the application.

**Figure 1:**
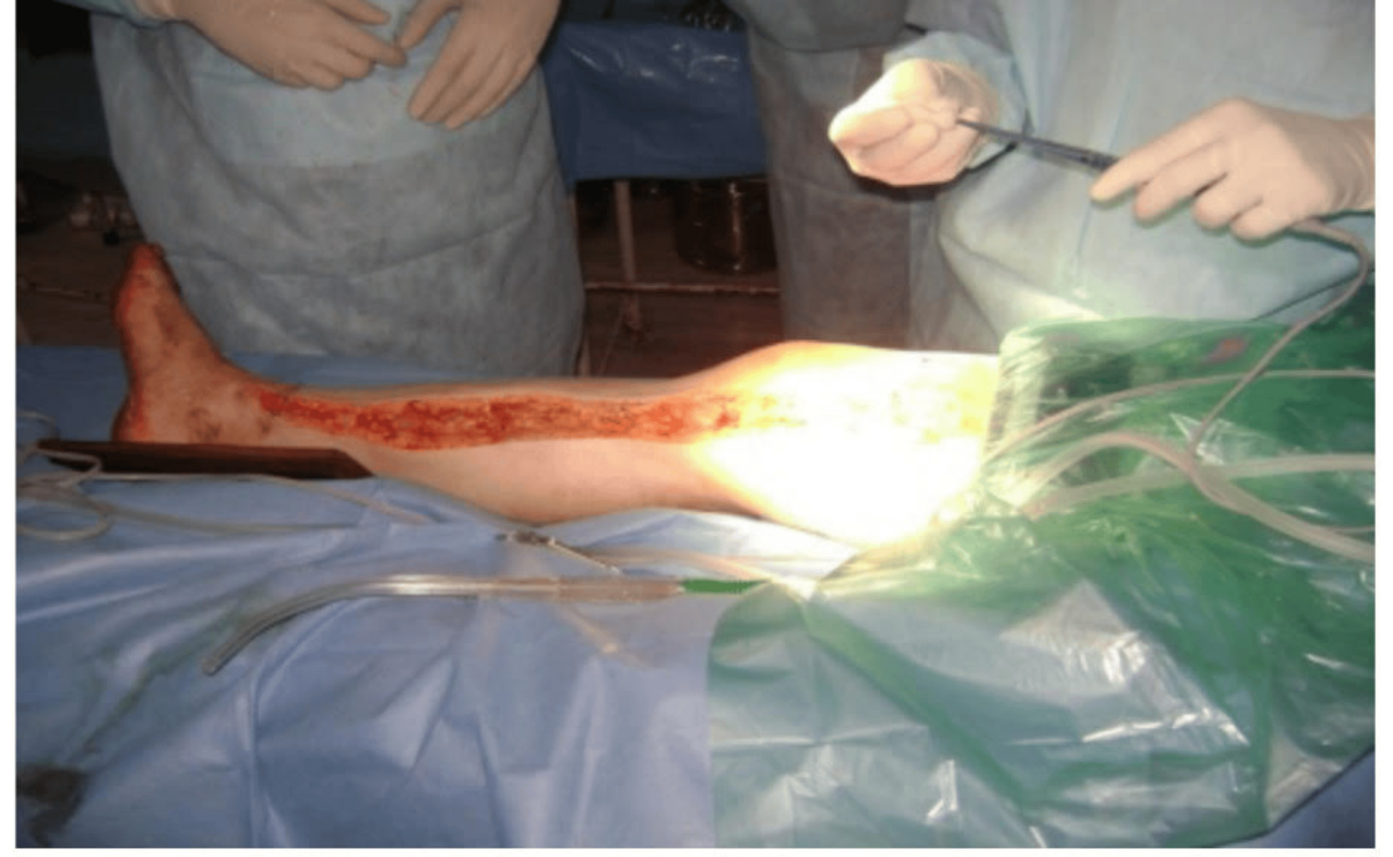
Cadaveric skin usage on burn patient: a case of burns

**Figure 2:**
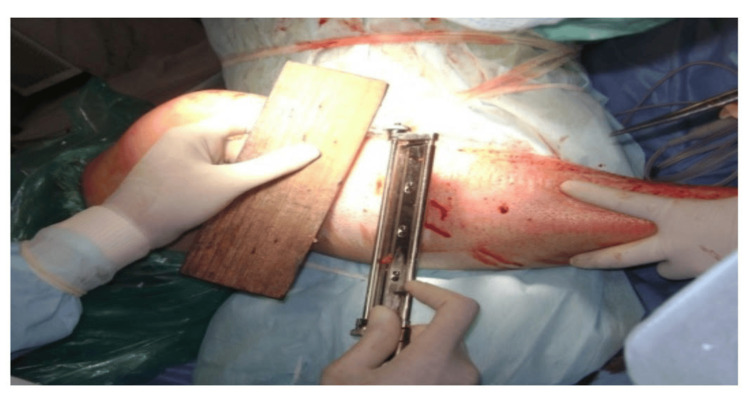
Cadaveric skin usage on burn patient: tangential excision being done

**Figure 3:**
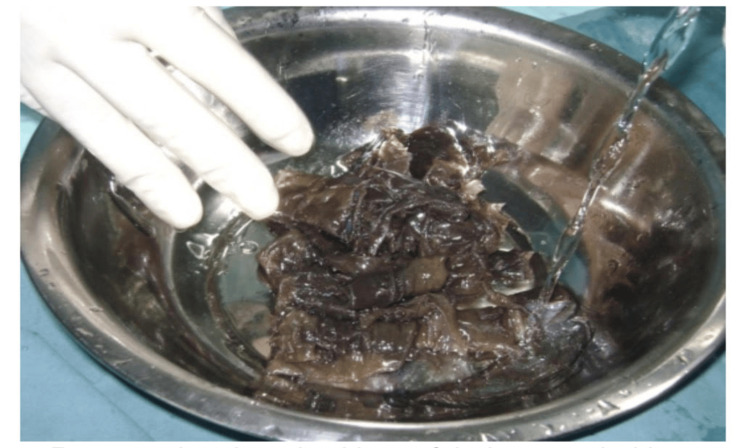
Cadaveric skin usage on burn patient: preserved cadaveric skin graft being emptied into sterile container

**Figure 4:**
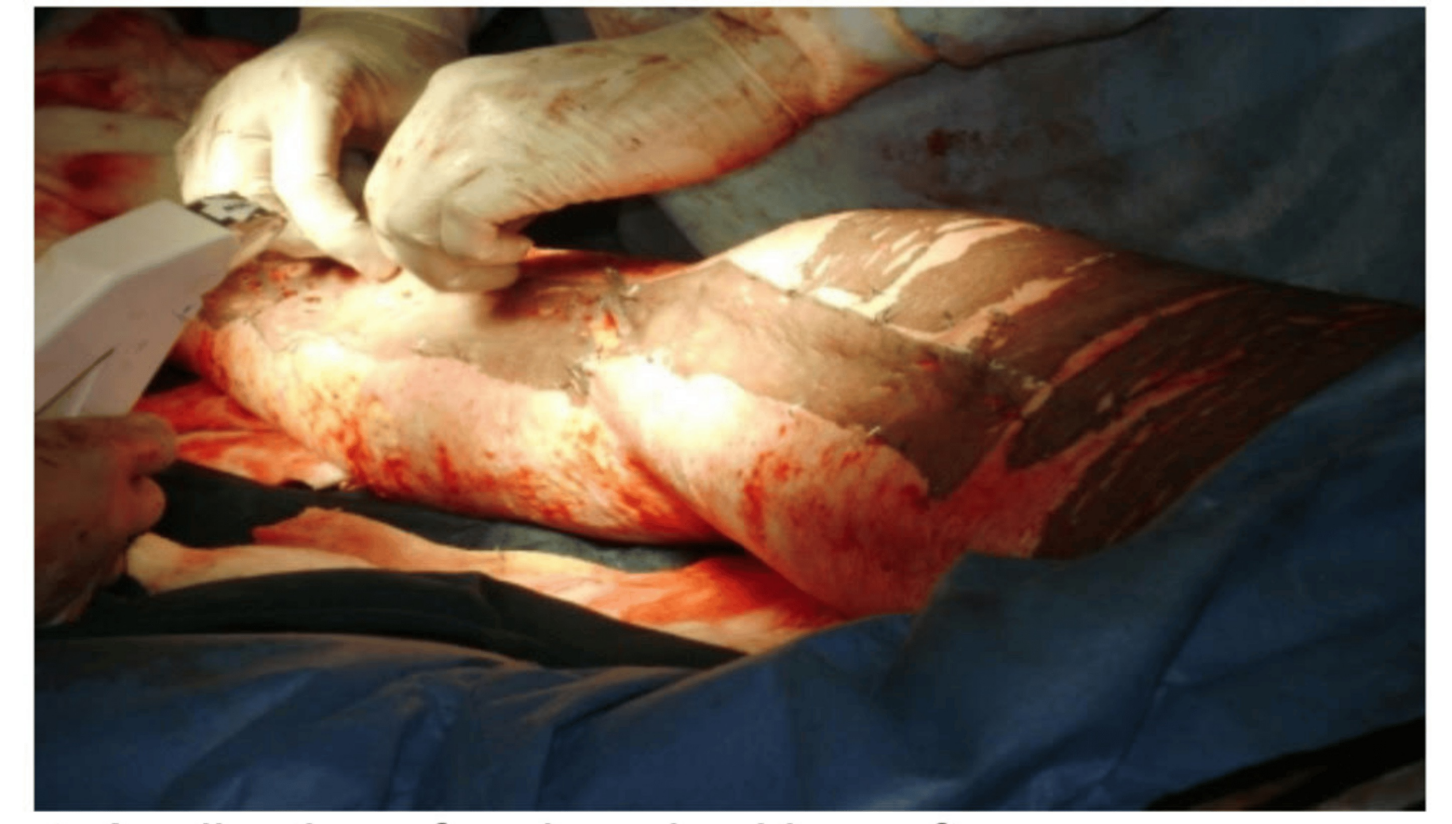
Cadaveric skin usage on burn patient: application of cadaveric skin graft

**Figure 5:**
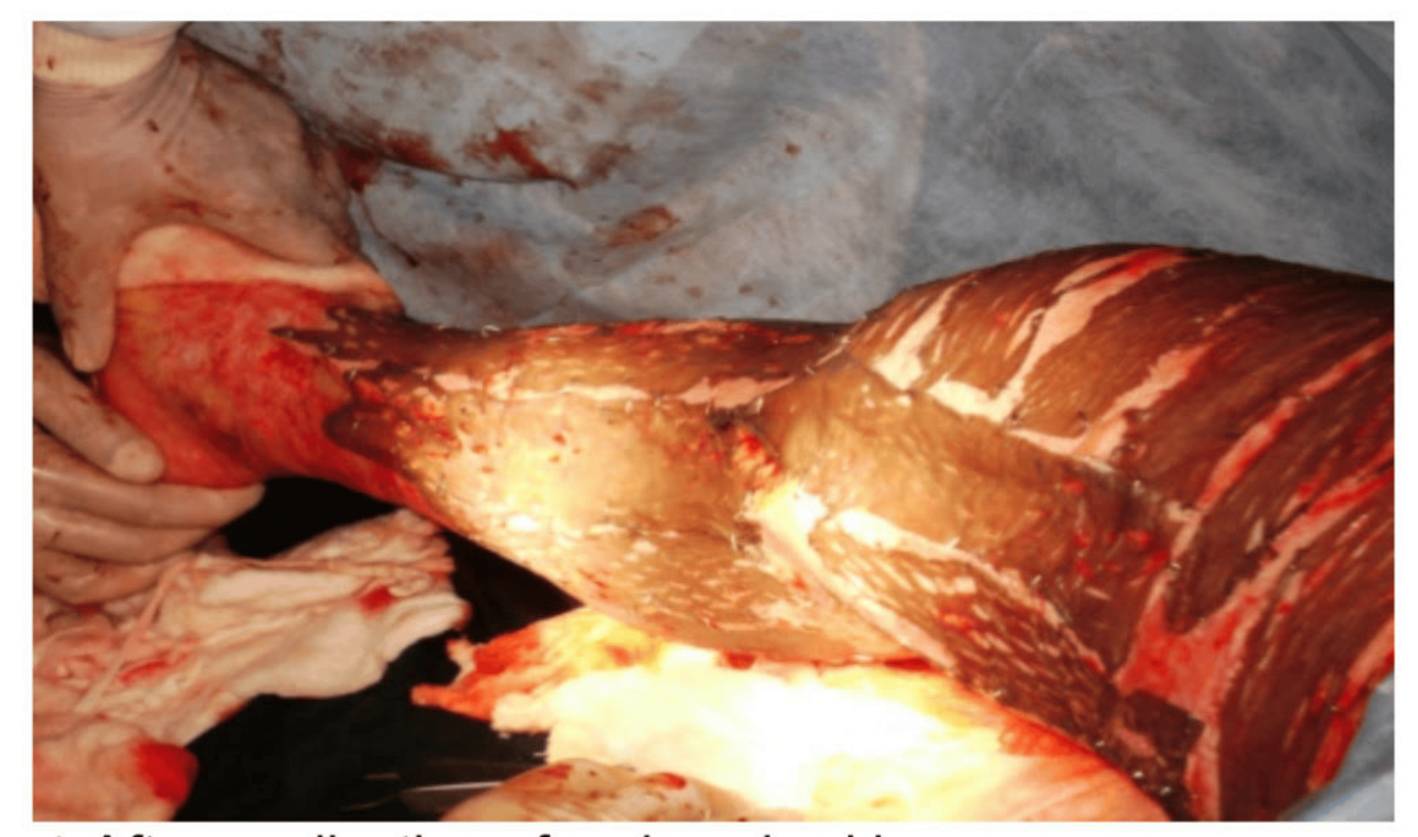
Cadaveric skin usage on burn patient: after application of cadaveric skin

**Figure 6:**
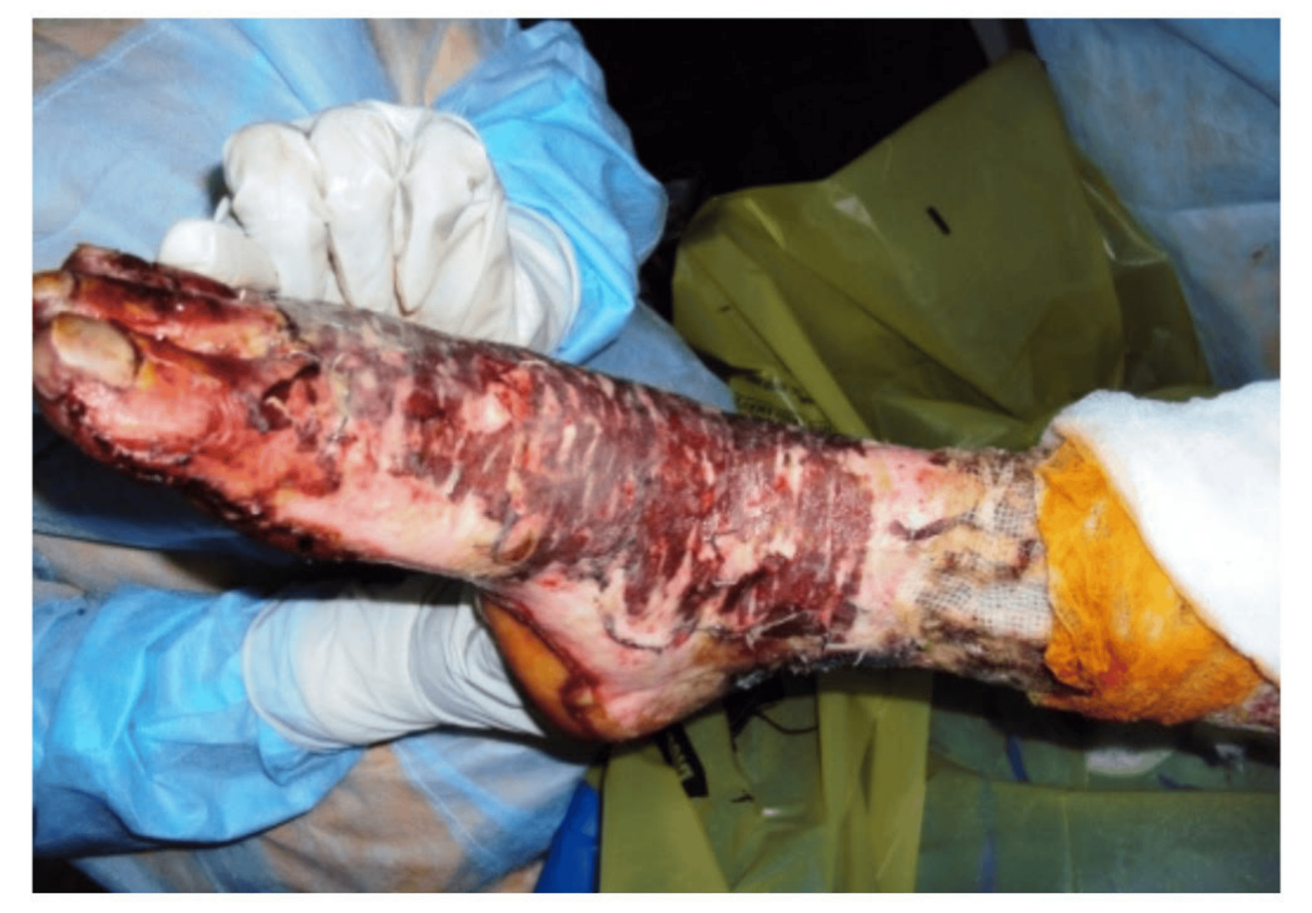
Cadaveric skin usage on burn patient: uptake of the cadaveric skin graft noted on day 10

**Figure 7:**
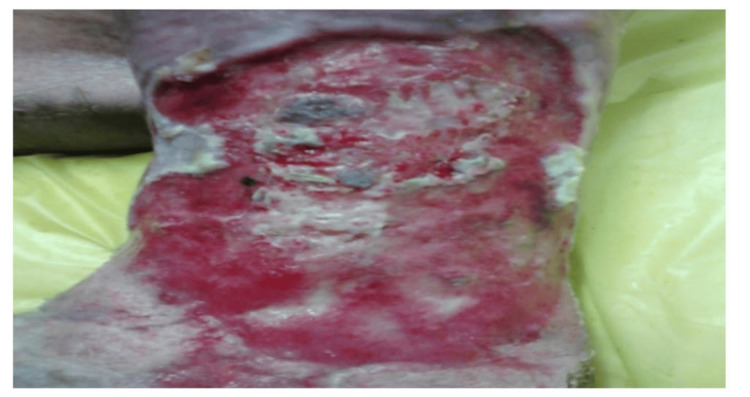
Cadaveric skin usage on a non-healing ulcer: non-healing ulcer on the left lower limb

**Figure 8:**
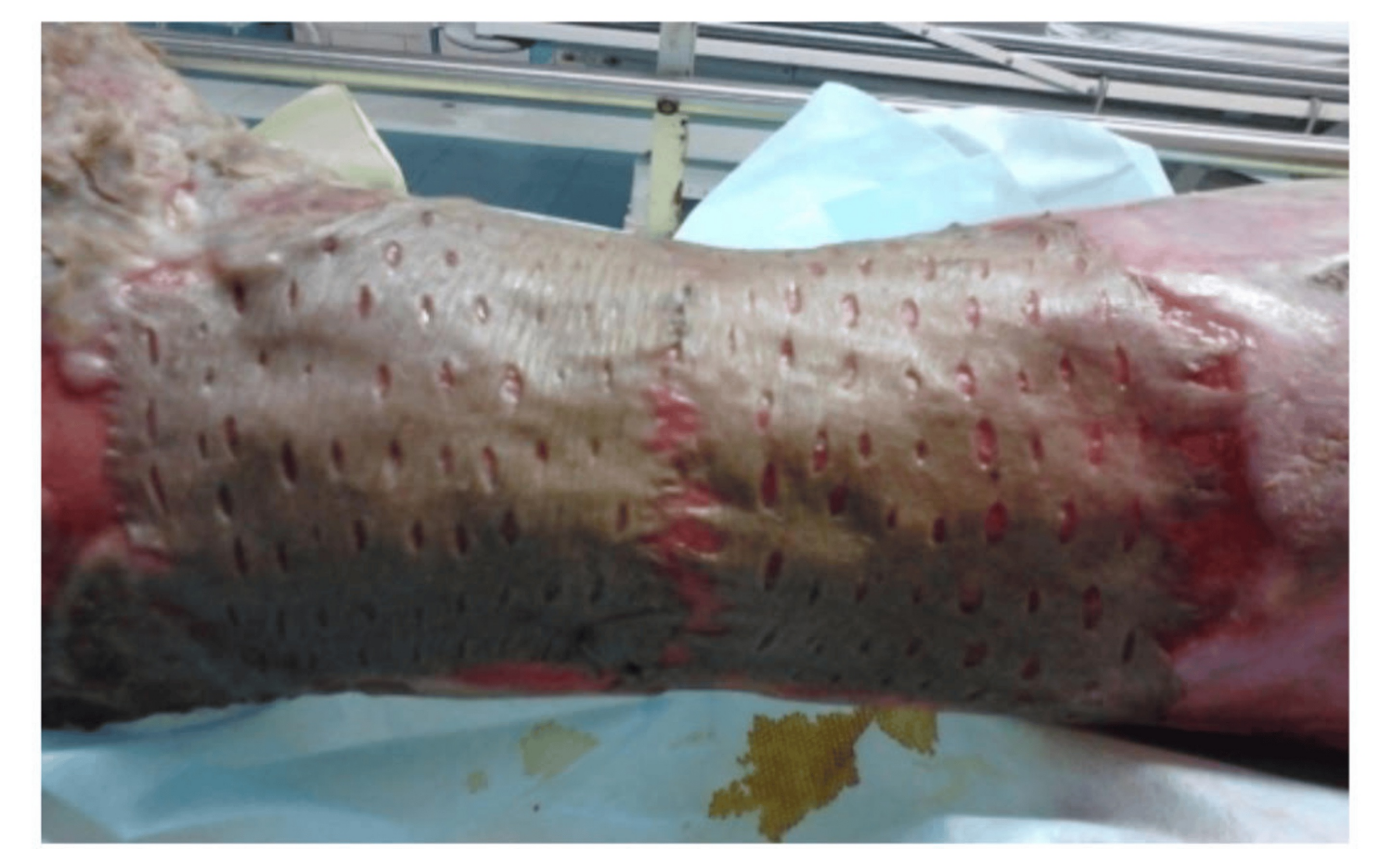
Cadaveric skin usage on a non-healing ulcer: application of cadaveric skin graft after wound debridement

**Figure 9:**
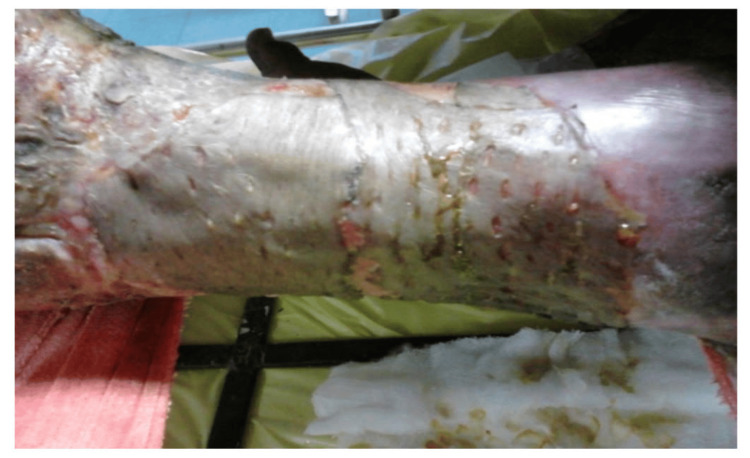
Cadaveric skin usage on a non-healing ulcer: uptake of cadaveric graft after five days

**Figure 10:**
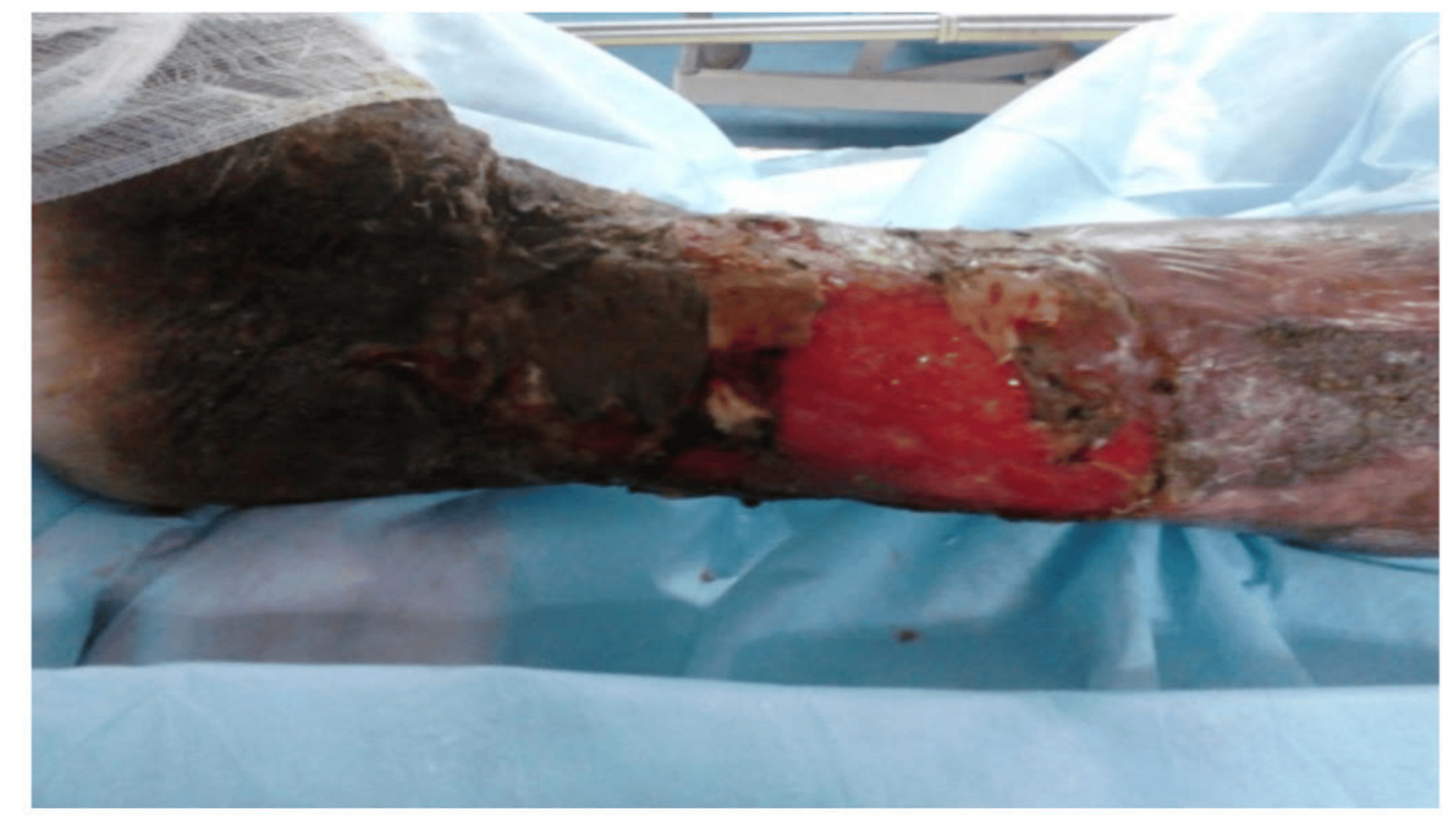
Cadaveric skin usage on a non-healing ulcer: inspection of wound on day 10

**Figure 11:**
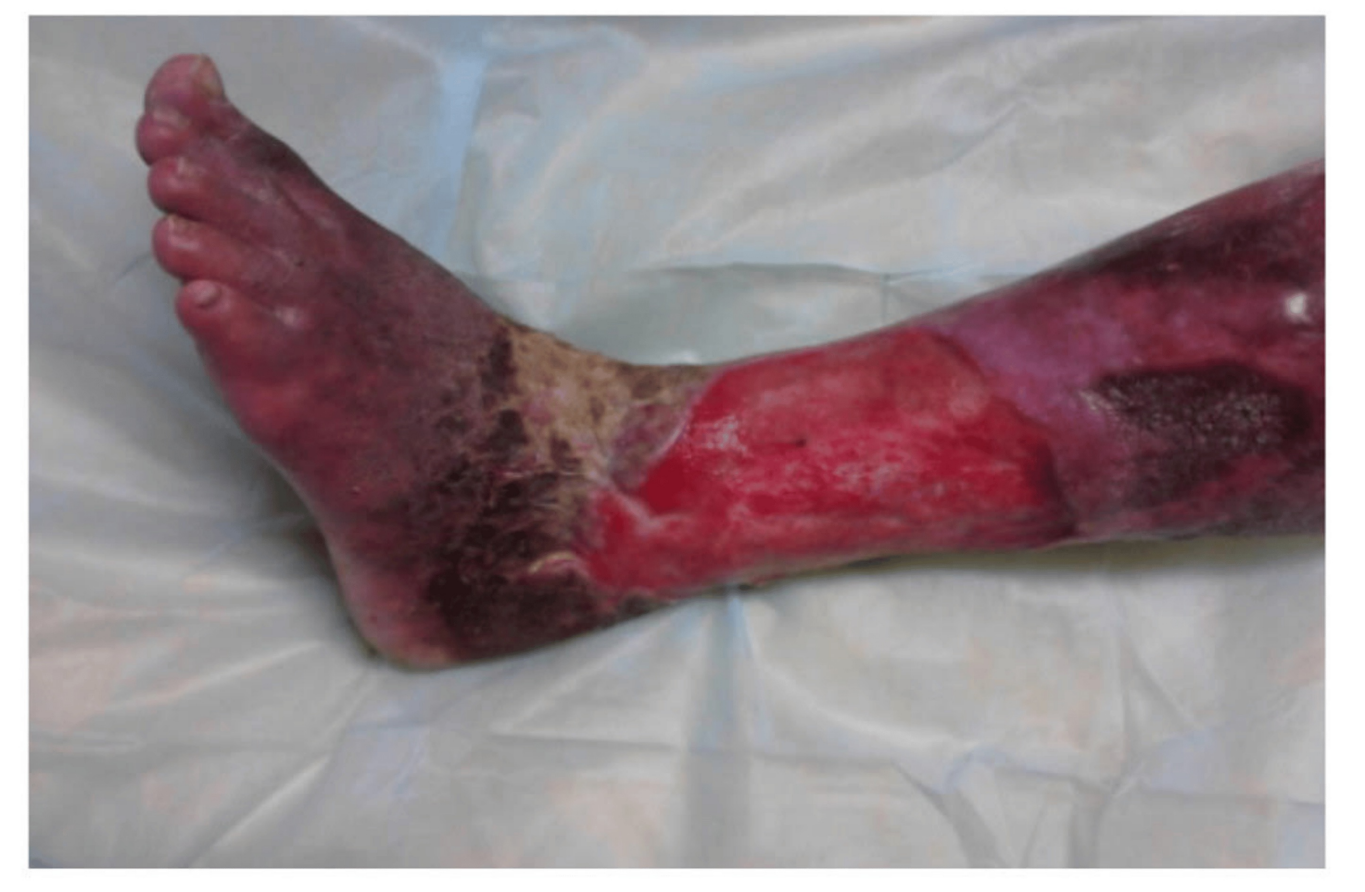
Cadaveric skin usage on a non-healing ulcer: inspection of wound after 14 days showing healthy granulation bed

Uptake of graft

The uptake of the grafts in cases of non-healing ulcers was 100% in both instances. For burn patients, the majority showed over 90% uptake of the graft. The detailed uptake of the graft in burn cases is presented in Table 2. On day five, four (16%) patients had 100% graft uptake, and 21 (84%) had over 90%. By day seven, no patients had 100% uptake, but 17 (68%) had over 90%, and six (16.3%) had over 85%. On day 10, no patients had 100% uptake, seven had over 90%, eight had over 85%, and seven (28%) had over 80%. Additionally, there were two (8%) deaths reported by day seven and three (12%) more by day 10.

**Table 2:** Uptake of graft in cases of burns

Uptake of graft	Day 5 (No of cases)	Day 7 (No of cases)	Day 10 (No of cases)
100%	4	Nil	Nil
>90%	21	17	7
>85%	Nil	6	8
>80%	Nil	Nil	7
Death	Nil	2	3
Total	25	25	25

Outcome and burn extent

The graft uptake was found to render the wound bed healthy and ready for permanent skin cover. Table 3 outlines the distribution of recipients according to the extent of burns and their outcomes. There were eight (32%) recipients for patients with up to 30% total body surface area (TBSA) burns, all of whom had more than 90% graft uptake and survived. In the 31-50% TBSA burn group, 12 (48%) patients received grafts, with nine (75%) survivors and three (25%) deaths. Among those with 51-70% TBSA burns, three (12%) patients received grafts, with one (33.33%) survivor and two (66.67%) deaths. For patients with burns exceeding 70% TBSA, there were two (8%) recipients, both of whom succumbed to their injuries. Overall, 18 out of the 25 patients with burn injuries survived, resulting in a survival rate of 72%, while seven (28%) patients died.

**Table 3:** Burn extent, allograft utilization, and outcome

Burn Extent (With TBSA)	Number of recipients	Uptake	Number of survivors	Number of deaths
Up to 30%	8	>90%	8	Nil
31-50%	12	>90%	9	3
51-70%	3	>90%	01	2
>70%	2	>90%	Nil	2
TOTAL	25	>90%	18	7

Demographics and outcomes

The youngest patient was four years old, and the age range of recipient patients was from four to 70 years. Among the recipients, 12 (48%) were males, and 13 (52%) were females. Seven deaths were among the 23 (30.43%) recipients of allografts. The causes of death were overwhelming sepsis in five (71.4%) cases and multi-organ failure following primary excision in two (28.7%) cases. The survivor with the largest burn extent had sustained 65% TBSA burns.

Multiple operations

Nine (36%) burn patients required more than one operation for tangential excision and application of cadaveric skin grafts at different burn sites each time. This repeated intervention was necessary to manage the extent of their injuries effectively.

## Discussion

Cadaveric skin has long been the standard biomaterial for temporary skin replacement in patients with extensive burns [8]. To date, there is no better alternative to the biological properties of cadaveric skin [9]. Many studies have reported that cadaveric skin has dermal elements that modulate the nature of the wound bed to create a milieu ideal for keratinocyte growth and skin reconstitution [10-14]. Although cadaveric skin traditionally offers temporary coverage for large wounds in burn patients, recent investigations suggest additional applications [15]. In the current study, human cadaveric skin was harvested and used as temporary biological coverage after adequate debridement in wounds of chronic non-healing ulcers and burns. There were significant issues in obtaining the willingness of the next of kin for cadaver skin donation. Firstly, the immediate outburst of emotions following the death of a loved one often hindered rational decision-making, leading to the refusal of skin donation. Secondly, there was a considerable lack of awareness among the patients and their next of kin, many of whom were from rural backgrounds, illiterate, or simply unaware of cadaveric skin donation as a concept. Superstition also played a major role; superstition is a widespread social issue affecting people from different educational backgrounds in India. Many of these beliefs are deeply rooted in tradition and religion; thus, introducing any change often faces opposition. Common beliefs encountered during the study included the notion of life after death, where it is believed that the individual will be born with a bodily disfigurement if their skin is donated. Additionally, some believed that the soul of the deceased existed in some form for about eleven days, and failing to give due respect to the body could bring calamity to the family.

Social stigma also contributed to the reluctance of patients and their next of kin. Many were extremely discontent with the idea of skin donation due to the fear of external deformation after harvesting. Despite detailed explanations of the process, they remained apprehensive about societal and familial judgments. Furthermore, religious issues were significant; while most major religions are favorable towards organ donation and accept individual decision-making rights, there was unwillingness among some patients and next of kin to donate skin due to the requirement of bathing the body before burial or cremation, which would expose the donor parts. The results of cadaveric skin application in this study were similar to those reported by Snyder [16], who found cadaveric skin advantageous in preventing wound desiccation, controlling infection, and substantially reducing pain in treating non-healing ulcers. Tzeng et al. [17] described their experience using cadaveric skin allografts as temporary biological coverage in chronic ulcers, diabetic foot ulcers, necrotizing fasciitis, and acute traumatic wounds. They concluded that patients with necrotizing fasciitis required the largest number of debridements and more cadaveric skin allografts. After clinical determination of engraftment one week post-allograft, histological examination of skin samples in 15 cases revealed migration of epithelia from the patient's skin to the cadaver skin surface and granulation tissue presence at the base of the cadaver skin. All wounds showed good wound-bed preparation post-transplantation and could eventually be resurfaced with a skin autograft.

Carucci et al. [18] suggested that cadaveric skin might be useful in stimulating granulation tissue after Mohs microscopic surgery for treating skin cancers involving the nose. The current trend in burn wound care has shifted from merely achieving satisfactory survival rates to improving the long-term form and function of healed burn wounds and the quality of life. This change has driven the emergence of various skin substitutes in managing burn injuries. Timely restoring skin protective functions is crucial for successfully treating burn victims with varying skin damage. Conventionally, autologous split or full-thickness skin grafts have been recognized as the best definitive burn wound coverage, but they are limited by the available sources, especially in major burns. Concerns about donor site morbidities, including additional wounds and scarring, also accompany autograft application. Dermal or bi-layered skin substitutes, hydrocolloids, and composite dressings are typically used by clinicians for temporary skin coverage [19]; however, this study's findings suggest that cadaveric skin can achieve the same purpose.

All wounds included in this study were eventually closed using a skin autograft due to adequate granulation in the wound bed, except for those burn cases where patients succumbed to their illness. The study's limitations include a small sample size of 44 cadaveric skin graft harvests and 27 applications, which may not represent the broader population and affect generalizability. Being a single-center study, results are influenced by unique institutional practices, necessitating multi-center studies for broader validation. Selection bias is present due to specific inclusion and exclusion criteria, potentially overlooking other relevant experiences. Focusing on immediate outcomes without long-term follow-up data limits understanding of graft durability and complications. Significant barriers to consent, such as lack of awareness and cultural objections, were identified but not addressed with effective strategies. The study did not extensively compare cadaveric grafts with synthetic alternatives, limiting insights into best practices. Potential confounding factors, ethical considerations, and technical procedure variability were not thoroughly explored. Lastly, reliance on retrospective data collection introduces reporting bias, highlighting the need for prospective studies with standardized data methods. Addressing these limitations in future research could provide a more comprehensive understanding of the use and impact of cadaveric skin grafts in burn care and other wound management applications.

## Conclusions

The study underscores the challenges and outcomes of cadaveric skin graft harvest and usage in tertiary care. Among the deaths recorded, significant barriers to skin donation included septicemia, skin changes, late reporting, young age, medico-legal issues, and positive viral markers. A notable number of next of kin refused consent, indicating a need for better awareness and education on skin donation. Despite these challenges, cadaveric skin was successfully harvested in a few cases, with a fraction of deaths identified as potential donors. Donors were mainly older males, with the highest proportion in the 71-80 age group. The procurement process was efficient, with most skin harvested within three to four hours post-mortem. Cadaveric skin grafts were used in several cases, primarily for burns. Graft uptake was monitored over 10 days, showing high success rates, especially in non-healing ulcers. In burn cases, the uptake was generally high, though some patients experienced graft failure or death. Cadaveric skin facilitated wound bed preparation, leading to successful autograft closure in surviving patients. The survival rate among burn patients was notable, with deaths mainly due to sepsis and multi-organ failure. The study highlights the vital role of cadaveric skin in managing extensive wounds, particularly burns, and emphasizes the importance of timely consent and procurement. The findings suggest that increasing community awareness and addressing superstitions and social stigmas are crucial for improving donation rates. The effective use of cadaveric skin demonstrated its potential in preparing wound beds for permanent skin coverage, contributing to better patient outcomes.
